# Study of anticancer, antimicrobial, immunomodulatory, and silver nanoparticles production by Sidr honey from three different sources

**DOI:** 10.1002/fsn3.1328

**Published:** 2019-12-09

**Authors:** Hamed A. Ghramh, Essam H. Ibrahim, Mona Kilany

**Affiliations:** ^1^ Research Center for Advanced Materials Science (RCAMS) King Khalid University Abha Saudi Arabia; ^2^ Unit of Bee Research and Honey Production Faculty of Science King Khalid University Abha Saudi Arabia; ^3^ Biology Department Faculty of Science, King Khalid University Abha Saudi Arabia; ^4^ Blood Products Quality Control and Research Department National Organization for Research and Control of Biologicals Cairo Egypt; ^5^ Department of Microbiology National Organization for Drug Control and Research (NODCAR) Giza Egypt

**Keywords:** AgNPs, anticancer, antimicrobial, *Apis mellifera*, Sidr honey, splenic cells

## Abstract

Sidr honey is used as food and medicine in many countries. Study of immunomodulatory and anticancer activity of Sidr honey did not tested before. The aim of this work was to study the anticancer activity and immunomodulatory as well as antimicrobial potential of Sidr honey and its synthesized silver nanoparticles (AgNPs). Sidr honey from three sources (two from Kingdom of Saudi Arabia (KSA) and one from Pakistan) was diluted to 20% and tested for its biological activities and to synthesize AgNPs. The results demonstrated that honeys could produce AgNPs (spherical shape), modulated the growth of normal splenic cells, and have antimicrobial activities. Sidr honey has anticancer activity against HepG2 but not Hela cells. Sidr honey can be used as antimicrobial agent, but can be used as anticancer agent with care as it stimulated cell growth of some lines (e.g., Hala) and inhibited another (e.g., HepG2).

## Graphical abstract

Sidr honey is used as food and medicine in many countries. Sidr honey from three sources (two from Kingdom of Saudi Arabia (KSA) and one from Pakistan) was examined. The results demonstrated that honeys could produce AgNPs, modulated the growth of normal splenic cells, have antimicrobial activities and anticancer activity against HepG2 but not Hela cells.

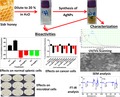

## 1 INTRODUCTION

In the market, there are wide varieties of honey (e.g., Manuka, Pasture, Jelly bush, Sidr [*Ziziphus* *spina‐christi*], Sumra, and Jungle) available, and these varieties are due to components gathered from different botanical sources. In reality, honey was used not only as food, but also as a traditional medicine and also has other several uses. Honey can be defined as the natural sweet material produced by an insect (bee) called *Apis mellifera* after collection of nectar of plants and other sources, and combining with specific materials produced by the bee. This finally produced material is deposited, dehydrated, stored, and left in the honeycomb to ripen and mature. Bees forage different plants in the same trip, and as a result, honey is always a mixture of different sources; therefore, no honey is completely similar to another honey (Nouvian, Hotier, Claudianos, Giurfa, & Reinhard, 2015). Generally, honey contains about 80% carbohydrates (35% glucose, 40% fructose, and 5% sucrose) and the rest (20%) is water with some other active biomolecules (e.g., amino acids, vitamins, minerals, enzymes, organic acids, flavonoids, and phenolic compounds) (Finola, Lasagno, & Marioli, 2007; Yücel & Sultanoǧlu, 2013).

Health benefits of honey depend on its quality and purity derived from the collected natural substances. Monofloral honey is defined as that type of honey which has a high value in the marketplace due to its distinctive flavor and other attributes resulted being predominantly from the nectar related to one plant species (Cotte, Casabianca, Chardon, Lheritier, & Grenier‐Loustalot, 2004). Sidr monofloral honey is found in the desert areas of Yemen, Saudi Arabia, and Pakistan's Potohar region (Al‐Waili, Salom, Butler, & Al Ghamdi, 2011).

Honey has the power to kill microorganisms, and this power is attributed to the high osmolarity and pH, hydrogen peroxide, as well as the phytochemical nature of honey (Molan, 2015). The antimicrobial potential depends on several factors like the type of honey, geographical location, and the botanical nature (Jull et al., 2015). It was reported that honey has an inhibitory effect against about 62 species of bacteria (aerobes and anaerobes, gram positives and negatives) (Hussain et al., 2015; Patton, Barrett, Brennan, & Moran, 2006). Sidr honey is widely used as a medication to treat liver diseases, ulcers of the stomach, lung infections, malnutrition consequences, digestion problems, constipation, infections of eyes, infections following burns, wounds and surgery, and general health and vitality. Sidr honey is known to have a strong antioxidant and antibacterial activities (Alandejani, Marsan, Ferris, Slinger, & Chan, 2009). Saudi market has numerous honey kinds (produced locally and imported). Some of them are used as folk medicine.

Cancer is one of the major scaring diseases to human. Treatment using chemotherapy is the widely used approaches to treat, but long‐term use of this technique may lead to drug resistance. Some workers (Ma, Dong, & Ji, 2010; Sarkar, Banerjee, & Li, 2007) reported resistance to anticancer agents such as including doxorubicin, camptothecin, cisplatin, 5‐fluorouracil, and taxol. Because of this resistance and bad side effects of chemotherapeutic agents, search for safer and effective drugs is mandatory. Honey has several bioactive molecules such as caffeic acid, caffeic acid phenethyl ester, and flavonoid glycons which have been shown to have inhibitory effects on tumor cell division (Rao et al., 1993). Honey was reported to have a moderate antitumor and antimetastatic effects in tumors of some strains of mouse and rat (Gribel' & Pashinskiĭ, 1990). Bee honey was shown to inhibit bladder cancer (Swellam et al., 2003) and potentiate the antitumor effects of chemotherapeutic drugs (Saunders & Wallace, 2010).

Nobel metal nanoparticles such as gold and silver got an high level of interest because of their multipurpose applications in several fields like biology, medicine, industry, etc. (Yokoyama & Welchons, 2007). The physiochemical characteristics of silver nanoparticles (AgNPs) made it point of interest for many researchers (Sharma, Yngard, & Lin, 2009). Nanoparticles can be prepared chemically and physically (Hanžić, Jurkin, Maksimović, & Gotić, 2015; Maleki, Simchi, Imani, & Costa, 2012; Okitsu, Yue, Tanabe, Matsumoto, & Yobiko, 2001), but green synthesis using plants (He et al., 2018; Kumar & Yadav, 2009; Makarov et al., 2014), yeast, bacteria, and fungi (Singh, Kim, Zhang, & Yang, 2016) now more used because these methods are nontoxic, clean, and eco‐friendly. AgNPs have many biological properties such as anticancer, antimicrobial, antifungal, antiviral, anti‐inflammatory (He et al., 2018; Jeyaraj et al., 2013; Monteiro et al., 2012; Wong & Liu, 2010; Zhang, Liu, Shen, & Gurunathan, 2016), anti‐parasite (Marimuthu et al., 2012), and insecticidal potentials (Moorthi, Balasubramanian, & Mohan, 2015). In addition, silver nanoparticles have been used in industry like in paints, detergents (Gottesman et al., 2011), clothing (Perelshtein et al., 2008), and pharmaceutical preparations (Martinez‐Gutierrez et al., 2010). Preparation of nanoparticles using plant extract is valuable due to the ease of preparation methods and with low biohazardous contents (He et al., 2017).

In this work, we tried to study the immunomodulatory and anticancer activity of Sidr honey that did not tested before. The power of the Sider honey to synthesize nanoparticles and the antimicrobial activity were studied too. The results showed that the three types of Sidr honey have anticancer activity and immunomodulatory potentials as well as antimicrobial potential. Sidr honey could synthesize silver nanoparticles (AgNPs).

## 2 MATERIALS AND METHODS

### 2.1 Honey samples collection and preparation

In this study, 3 honey samples were collected and categorized as shown in Table 1. Samples were labeled and stored at 4°C till used in biological activity studies. Honey samples were diluted at 20% in distilled water and used fresh every time when used.

**Table 1:** Codes and types of collected honey samples

Sample code	Honey type	Honey bee species
H1	Sider (Rijal Ulma, Saudi Arabia)	*Apis mellifera jemenitica*
H2	Sider (Rijal Ulma, Saudi Arabia)	*Apis florea*
H3	Sider (Pakistani)	*Apis mellifera ligustica*

### 2.2 Biosynthesis of silver nanoparticles

Honey samples were used to synthesis silver nanoparticles (AgNPs) following the method shown by Ghramh, Al‐Ghamdi, Mahyoub, and Ibrahim (2018). In brief, 1 ml 20% honey was added to 99 ml 1 mM AgNO_3_ solution in an Erlenmeyer flask. The pH of the mixture was raised until the color change occurred.

### 2.3 Characterization

All characterization methods for AgNPs prepared by honey samples and active biomolecules found in honey samples before and after the synthesis of AgNPs were done following the same methods and instruments described by Ibrahim, Kilany, Ghramh, Khan, and ul Islam (2018).

### 2.4 Antimicrobial potential test

Well diffusion assay was adopted according to Kilany (2016) using gram‐positive bacteria (*Bacillus subtilis*), gram‐negative bacteria (*Escherichia coli and Pseudomonas aeruginosa*), and the fungal strain *Candida albicans* as a model of fungus. The 6‐mm wells were aseptically bored into agar, and 40 µl of honey or honey containing AgNPs from each sample was aseptically pipetted into the wells. Penicillin (10 µg) was used as positive control.

### 2.5 Effects of different honey samples on normal rat splenic cell proliferation

Adult male Sprague Dawley rat weighing 239 g was kindly given by the animal house at King Khalid University. Single‐cell suspension at density of 4 × 10^4^/ml was prepared according to Algarni et al. (2019). A 100 μl culture media containing 20% honey or 20% honey containing AgNPs were added to 100 μl of the cell suspension (4,000 cells/well). Control untreated cell culture was included. Plates containing the cells were incubated for 72 hr at 37°C in 5% CO_2_ (CO_2_ Incubator, Memmert, Gmbh). Number of viable cells was measured using Vybrant® MTT Cell Proliferation Assay Kit (Thermo Fisher Scientific) (Ibrahim et al., 2018).

### 2.6 Anticancer activity test

The cell lines HepG2 and Hela were used to test the anticancer potential of honey and honey containing AgNPs. The cells were maintained and grown in supplemented minimal essential medium (MEM), fetal calf serum (10%), penicillin/streptomycin (100 U/ml/100 μg/ml), and l‐glutamine (2 mM) in the CO_2_ incubator. After reaching confluency, cells were trypsinized (2% trypsin‐EDTA) to prepare single‐cell suspension. Single‐cell suspension was adjusted to 1 × 10^5^/ml, and then 100 µl (10^4^ cells) was plated into each well of 96‐well plate and incubated overnight in the CO_2_ incubator. The medium in the plate was decanted and 200 µl media containing 20% honey and 20% honey containing AgNPs. The plate was incubated for an additional 24 hr in the CO_2_ incubator. The media in wells were replaced with a fresh 100 µl/well culture medium. The viability of the cells was determined the exact way as above.

## 3 STATISTICAL ANALYSIS

All data were expressed as the mean of three triplicates. Different concentrations of the extract and extract generated AgNPs differences were analyzed with one‐way analysis of variance (ANOVA) using SPSS (version 17). Differences of *p* ≤ .05 were considered to be statistically significant.

## 4 RESULTS AND DISCUSSION

### 4.1 Characterizations and sample analysis

**Figure 1:**
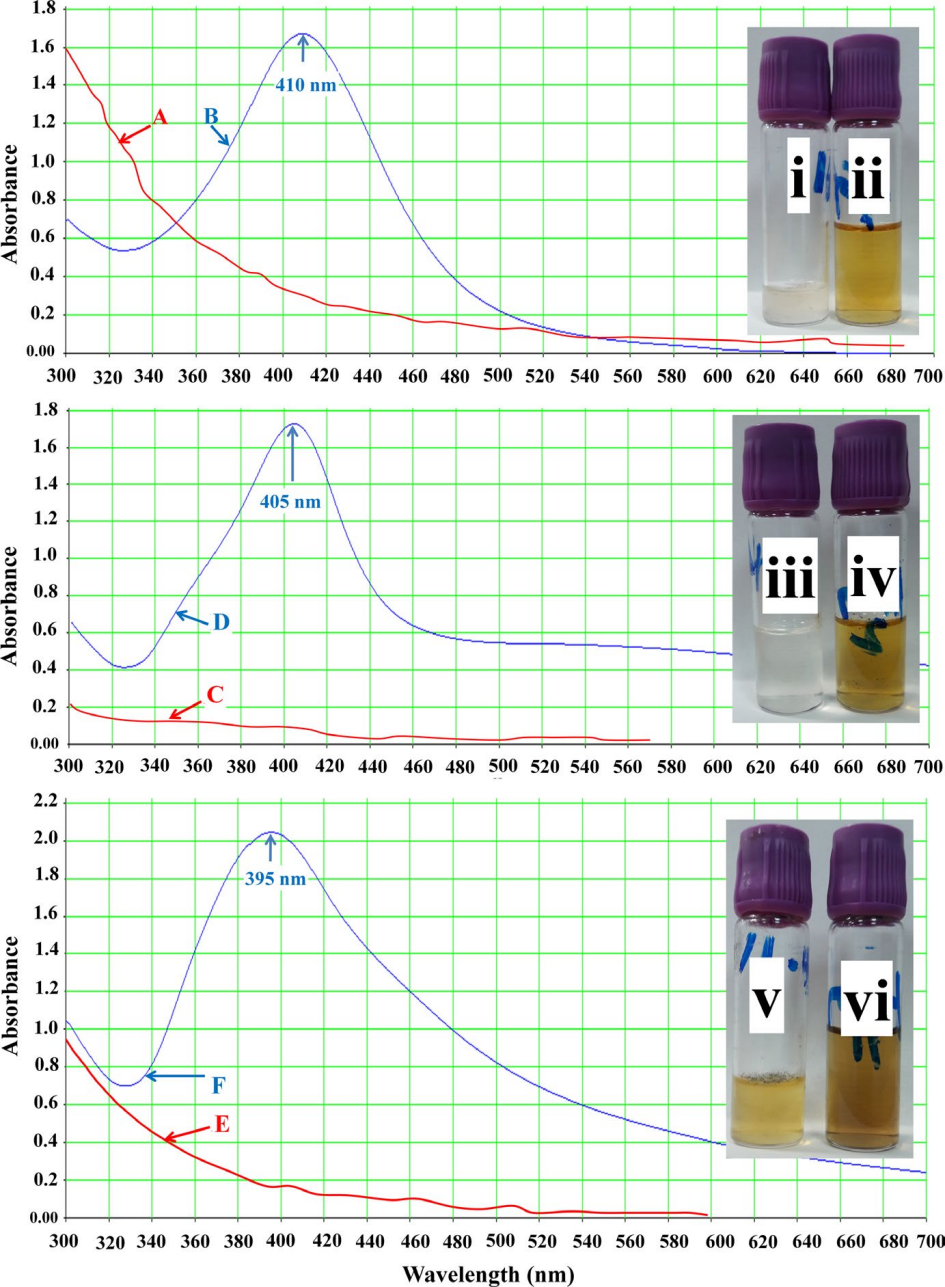
Silver nanoparticle synthesis by 20% Sidr honey. Where A, C, and E stand for color absorbance of H1, H2, and H3 alone, respectively; B, D, and F stand for color absorbance of H1, H2, and H3, respectively, after the synthesis of AgNPs; i, iii, and v stand for color of H1, H2, and H3, respectively, and ii, iv, and vi stand for color of H1, H2, and H3, respectively, after the synthesis of AgNPs

Diluted honey samples were mixed with silver nitrate to synthesize AgNPs. The change in color of the mixture was an indication of AgNPs synthesis where the color of the solution changed from pale yellow to brown and continued to dark brown (Figure 1i–vi). The degree of color change was pH‐dependent that enabled the visual monitoring by observation. The pH of the samples was 3.52, 3.59, and 3.25 for H1, H2, and H3, respectively. The color change was complete when the pH reached 9.

Both diluted honey (20%) before adding AgNO_3_ and honey after the complete color change to brown were scanned spectrophotometrically. Results indicated the formation of silver nanoparticles after treated with AgNO_3_. H1 showed a peak at 410 nm (Figure 1B), H2 at 405 nm (Figure 1D), and H3 at 395 nm (Figure 1F).

Honey is an extremely complex food product that has been reported to contain at least 181 different substances including proteins, enzymes, amino acids, minerals, vitamins, and polyphenols (Balasooriya et al., 2017; Philip, 2009). There is a possibility that sucrose, glucose, and proteins/enzymes play a part in the reduction process. The addition of NaOH, which consequently increased the pH of the solution, has an effect on the size of the nanoparticles produced. This probably due to the increased formation of gluconic acid from glucose as pH increased. Based on a number of literature studies, gluconic acid is formed from glucose because the base drives the opening of the glucose ring by abstraction of the α‐proton of the sugar ring oxygen. The Ag ions then oxidized glucose to gluconic acid and itself reduced to metallic Ag. However, the actual ingredients which are responsible for the reduction of the Ag ions still remain unknown and need further study.

**Figure 2:**
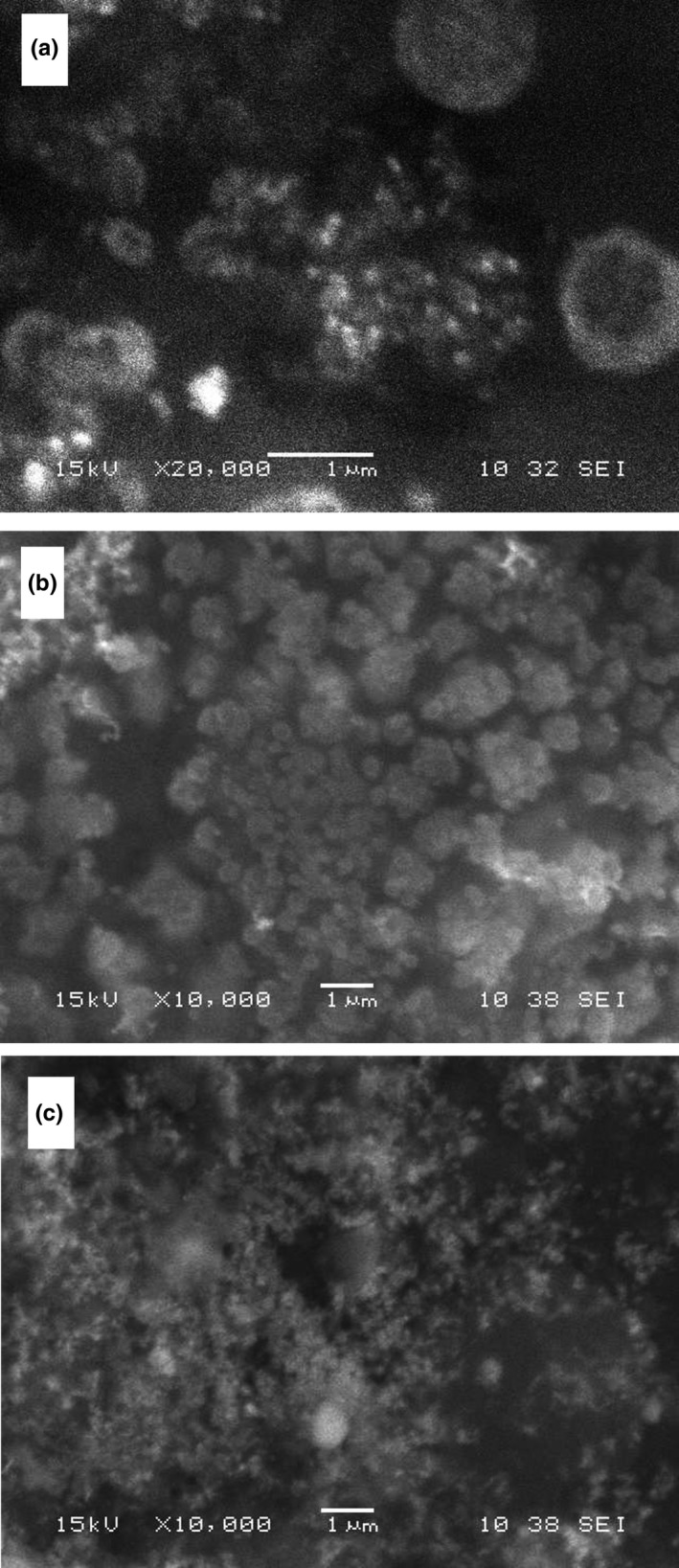
The SEM images showing the spherical silver nanoparticles produced by H1 (a), H2 (b), and H3 (c)

Scanning electron micrographs showed that the H1‐, H2‐ and H3‐synthesized AgNPs are spheres and of a size about 70–80 nm (Figure 2a), 80–90 nm (Figure 2b), and 50–60 nm (Figure 2c), respectively.

Mock, Barbic, Smith, Schultz, and Schultz (2002) using high‐resolution TEM images of silver nanoparticles with different sizes and the geometrical shape showed that at the surface plasmon resonance (SPR) peak range 410–500 nm the shape of the particles is spherical, whereas pentagons and triangular shapes are mostly formed at wavelengths from 500 to 700 nm. This observation strongly suggests that the Ag nanoparticles formed here were spherical.

### 4.2 Functional groups characterization

**Figure 3:**
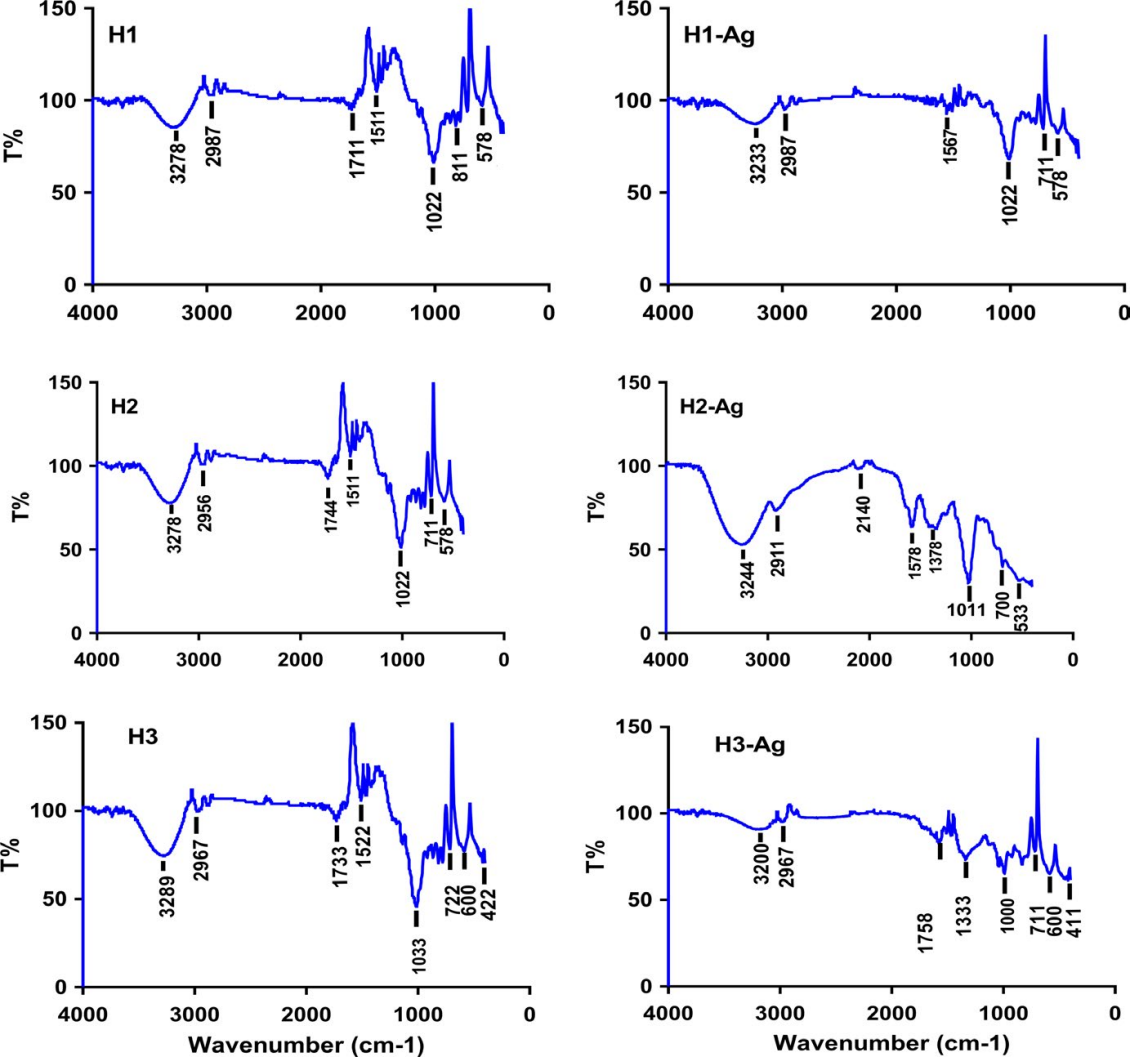
FTIR spectra of H1, H2, and H3. Where H1, H2, and H3 before and H1‐Ag, H2‐Ag, and H3‐Ag after the addition of AgNO_3_

FTIR spectroscopy is useful in probing the chemical composition of the surface of the silver nanoparticles and the local molecular environment of the capping agents on the nanoparticles. Figure 3 shows the FTIR spectra of honey and honey containing silver nanoparticles obtained in this study.

The bioactive compounds of honey 1 (H1) and the biosynthesized silver nanoparticles (AgNPs) were traced by FTIR spectrophotometer shown in Figure 3 (Figure 3, 4‐H1 and Figure 3, 4‐H1‐Ag). Totally, 7 peaks were obtained in the case of honey. The broadband appearing at 3,278 cm^−1^ is assigned for O–H stretching vibration indicating the presence of alcohol or phenol as a reducing agent. Weak bands at 2,987, 1,711, and 1,511 cm^−1^ are assigned to C‐H, C=O, and N‐O stretching vibration indicating the presence of alkane, aliphatic ketone, and nitro compound, respectively. The strong, intense peaks at 1,022, 711, and 578 cm^−1^ correspond to C‐O, C=C, and C‐Br stretching vibrations indicating the presence of vinyl ether, alkene, and bromocompounds. The result of this FTIR spectroscopic study confirmed that the red apple fruit extract has the ability to perform dual functions of reduction and stabilization of silver nanoparticles. FTIR of AgNPs showed 6 peaks which are merely similar to that obtained by honey indicating the presence of alcohol, alkane, aliphatic ketone, nitro compound vinyl ether, alkene, and bromo compounds. The noticed difference is that peaks indicating alcohol, nitro compound, and alkene decreased in intensity indicating the exploitation of these compounds in the reduction and capping of silver nanoparticles.

**Figure 4:**
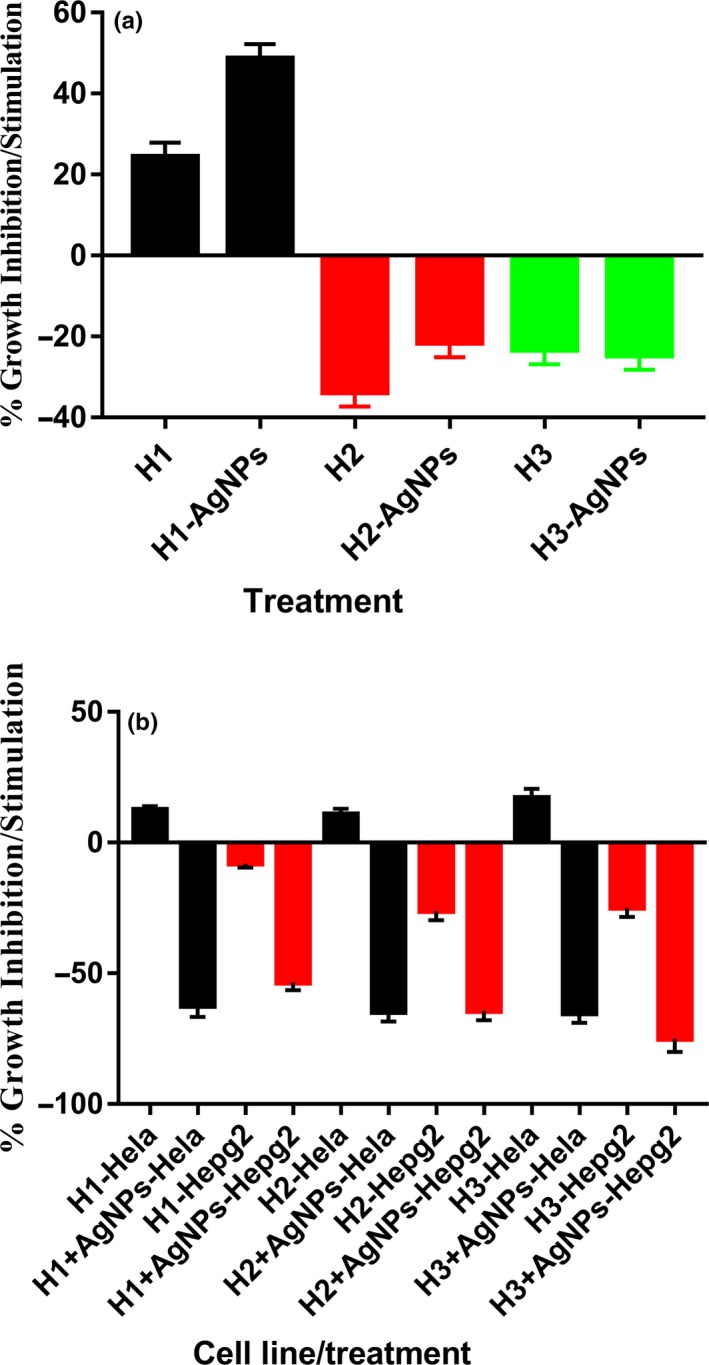
Effects of different honey (H1‐H3) treatments, alone or containing AgNPs, on normal rat splenic cells and cancer cell lines

The FTIR spectrum was documented for both honey (H2) and the biosynthesized silver nanoparticles (AgNPs) as shown in Figure 3 (Figure 3, 4‐H2 and Figure 3, 4‐H2‐Ag). The FTIR spectra of honey (H2) showed the characteristic 7 peaks of bioactive compounds. The broadband appearing at 3,278 cm^−1^ is assigned for O–H stretching vibration indicating the presence of alcohol as a reducing agent. Weak bands at 2,956, 1,744, and 1,511 cm^−1^ are assigned to C‐H, C=O, and N‐O stretching vibration indicating the presence of alkane, cyclopentanone, and nitro compound, respectively. The strong peaks at 1,022, 711, and 578 cm^−1^ correspond to C‐O, C=C, and C‐Br stretching vibrations indicating the presence of vinyl ether, alkene, and bromo compounds. On the other hand, FTIR of AgNPs showed 7 peaks, some of them similar to that obtained in honey spectrum meanwhile some new peaks arisen, such as weak band at 2,140 cm^−1^ may correspond to nitrile CΞN stretch or alkynyl CΞC stretch. Medium peak at 1,578 cm^−1^ assigned to C=C stretching of cyclic alkene. Another peak observed at 1,578 cm^−1^ assigned to C=O stretching vibrations of amide. Medium peak at 1,378 cm^−1^ may be attributed to C‐H bending due to alkane. So, these compounds produced as a result of the reduction of silver nitrate to silver nanoparticles. On the other hand, some peaks disappeared such as that corresponding to cyclopentanone, alkene, and bromo compounds, indicating that they are used in the reduction and stabilization process of silver nanoparticles.

The functional groups involved in honey 3 and the formation of AgNPs using FTIR spectroscopy were shown in Figure 3 (Figure 3, 4‐H3 and Figure 3, 4‐H3‐Ag). Representative spectra of both H3 and H3 containing AgNPs manifest absorption peaks in the region 3,500–500 cm^−1^. The broad peak around 3,289 cm^−1^ in the spectra indicates the existence of O‐H group of alcohol. Weak bands at 2,967, 1,733, 1,522, 1,033, 722, and 600 cm^−1^ are associated with stretch vibration of C‐H, C=O, C=C, N‐O, C‐O, and C‐Cl that correspond to alkane, ketone, alkene, nitro compounds, vinyl ether, and alkyl halide, respectively. After nanoparticle synthesis, the bands shifted to 3,200, 2,967, 1,758, 1,000, 711, and 600 cm^−1^ bands with lower intensity which could be assumed that they were used in the reduction and capping of silver nanoparticles. Appearance of a new strong peak around 1,333 cm^−1^ corresponding to C‐N stretching of aromatic amine as a byproduct of reduction process. On the other hand, disappearance of the band at 1,522 cm^−1^ means that alkene is exhausted in the reduction and stabilization of nanoparticles.

### 4.3 Antimicrobial susceptibility testing (AST)

The results of antibacterial activity of Sidr honey against gram‐positive bacteria (*B. subtilis*), gram‐negative bacteria (*E. coli and P. aeruginosa*), and the fungal strain *C. albicans* are shown in Table 2.

**Table 2:** Antimicrobial potentials of honey types alone and containing AgNPs

	Inhibition zone (mm)			
	*Escherichia coli*	*Pseudomonas aeruginosa*	*Bacillus subtilis*	*Candida albicans*
Honey 1	13.50 ± 0.20	11.90 ± 0.19	12.43 ± 0.21	12.5 ± 0.80
Honey 1 + AgNPs	20.12 ± 0.29	12.65 ± 0.16	11.82 ± 0.12	15.20 ± 0.29
Honey 2	10.9 ± 0.35	10.9 ± 0.35	10.9 ± 0.35	10.9 ± 0.35
Honey 2 + AgNPs	18.10 ± 0.39	11.26 ± 0.12	10.22 ± 0.11	10.10 ± 0.13
Honey 3	7.8 ± 0.22	7.8 ± 0.22	7.8 ± 0.22	7.8 ± 0.22
Honey 3 + AgNPs	15.10 ± 0.18	11.80 ± 0.14	11.60 ± 0.17	9.70 ± 0.09
Penicillin (10 µg)	12.60 ± 0.02	9.80 ± 0.09	10.70 ± 0.03	10.40 ± 0.17

Regarding the results of antimicrobial activity of different honey types against *B. subtilis*, *P. aeruginosa*, and *E. coli* and the fungal strain *C. albic*ans, it was clear that all honey alone at the 20% concentration showed inhibition of bacterial growth. H1, but not H2 and H3, showed bacterial growth inhibition effect more than that of the positive control (Penicillin 10 µg). Notably, addition of AgNPs to both H1 and H3 clearly increased their growth inhibition effect but did not regarding to H2. The inhibition of bacterial growth may be due to many factors as the osmotic effect of honey (Kwakman et al., 2010; Kwakman & Zaat, 2012; Voidarou et al., 2011), the presence of hydrogen peroxide (Nassar et al., 2012), nonperoxide substances (Mandal & Mandal, 2011), and volatile antibacterial substances (Boateng & Diunase, 2015; Olaitan et al., 2007). Also, Jeddar et al. (1985) evaluated the growth of various gram‐positive and gram‐negative bacteria in media containing various concentrations of honey, and they found that most pathogenic bacteria failed to grow in honey at a concentration of 40% or above. The pH of honey being between 3.2 and 4.5 is low enough to inhibit pathogen growth. But if this honey is diluted with other fluids, for example by body fluids, the pH will raise and would not lower that effectively can inhibit bacterial growth (Molan, 1992, 2002, 2015).

The enzyme glucose oxidase (bee‐origin) and the enzyme catalase (floral origin) play an important role in the biological activities of honey (White et al., 1963). Regarding glucose oxidase enzyme, as this enzyme concentration increases in honey, the ability to hydrolyze glucose producing hydrogen peroxide (H_2_O_2_) increases, resulting in higher oxidative stress on microbial growth. In contrary, the increase in catalase enzyme concentration, which destroy H_2_O_2_, will determine the final antimicrobial effects. The balance of these two enzymes determines, at least in part, the antimicrobial activity of the honey (Zainol et al., 2013).

Some researcher demonstrated that undiluted honey has inactive glucose oxidase (White et al., 1963), meaning that the action of H_2_O_2_ is minimal and the antimicrobial effects of honey depend mainly on the very high osmotic pressures coupled with the high acidity are the two main factors contributing to the antimicrobial properties (Kwakman & Zaat 2012; Zainol et al., 2013). But, if honey is diluted, glucose oxidase will get activated and utilize glucose to produce H_2_O_2_. In the current study, all honeys were diluted using sterile distilled water to get the glucose oxidase activated. In diluted honey, if the osmotic pressure is decreased as a result of dilution, the antimicrobial potentials will be referred to the pH value and peroxide activity. In addition, some other components in the diluted honey can contribute to its antimicrobial activities that may include phenolic compounds, flavonoids, antibacterial peptides, methylglyoxal, methyl syringate, antibiotic‐like derivatives, and other components present in trace amounts (Jaganathan & Mandal, 2009; Mandal & Mandal, 2011). Others (AL‐Waili et al., 2013) concluded that, regarding that geographical areas and plant origins, all honey may show antimicrobial activities despite considerable variation in their composition.

### 4.4 Effects of different honey samples on normal rat splenic cell proliferation

Until now, the immunomodulatory effects of Sidr honey are relatively unknown. Therefore, in this study, we investigated the effects of Sidr honey collected from three different geographical locations on immune function and antitumor activity in vitro.

The proliferative/antiproliferative potentials in the tested honeys were examined using normal rat splenic cells (Figure 4a). Treatment with 20% H1 leads to growth stimulation of normal splenic cells. This growth stimulatory effect significantly (*p* < .001) increased when cells are treated with H1 containing AgNPs. In contrary, H2 and H3 inhibited the cell's growth. H2 nonsignificantly inhibited cell's growth more than H3. The degree of inhibition of H2 was nonsignificantly lowered when H2 contained AgNPs. H3 inhibited splenic cell's growth nearly the same as H3 containing AgNPs.

Some researchers (Tonks et al., 2003; Tonks, Cooper, Price, Molan, & Jones, 2001) reported that honey (Manuka) increased factors that decrease cell growth like IL‐1β, IL‐6, and TNF‐α production by monocytes through a 5.8 kDa protein. The expected mechanism by which the increase in these cytokines production is via TLR4 (Tonks et al., 2007). Others (Al‐Waili & Haq, 2004) reported that intake of honey (oral) augmented the production of antibodies in primary and secondary immune responses.

One of the explanations that honey lowers the cell growth is by arresting cell cycle (Tomasin & Cintra Gomes‐Marcondes, 2011). The components contained by honey (e.g., flavonoids and phenolics) are reported to block the cell cycle of many cell types (Jaganathan & Mandal, 2009; Lee et al., 2003; Pichichero, Cicconi, Mattei, Muzi, & Canini, 2010) in G0/G1 phase. This inhibitory effect exerted on the proliferation of cells directly follows the downregulation of several cellular pathways through tyrosine cyclooxygenase, ornithine decarboxylase, and kinase (Jaganathan & Mandal, 2009; Oršolić et al., 2010; Pichichero et al., 2010). Others showed that 3‐(4,5‐dimethylthiazol‐2‐yl)‐2,5‐diphenyl tetrazolium bromide (MTT) assay method confirmed that antiproliferative effect of honey is in a dose‐ and time‐dependent manner (Pichichero et al., 2010). Honey or its components mediate inhibition of cell growth due to its perturbation of the cell cycle (Oršolić et al., 2010; Pichichero et al., 2010).

Another explanation was shown by some workers (Duddukuri, Rao, & Athota, 2008) where they suggested that the inhibitory potential of honey may be due to the direct suppressive effect of honey on T‐cell proliferation. When adding silver nitrate to the honey, some biomolecules are consumed to produce silver nanoparticles as indicated earlier using FTIR analysis in this work. The inhibitory effects of honey nonsignificantly decreased when contained AgNPS. This is may be due to the use of some inhibitory biomolecules in nanoparticle formation. The consumption of these expected biomolecules gave the chance to stimulatory biomolecules to dominate in the medium, explaining the stimulatory behaviors of the honey when mixed with AgNPs.

### 4.5 Anticancer activity

The potentials of the three Sidr honeys to kill or stop cancer cell proliferation were tested using two cell lines (Figure 4b). All honeys did not show any anticancer activities against the Hela cell line. But, when honey contained AgNPs, anticancer activities were shown by all honeys. The anticancer activity against the Hela cell line of H1, H2 and H3 containing AgNPs was shown to be not significantly different. In contrary to the anticancer activity against Hela cells, H1, H2 and H3 showed anticancer potentials against the HepG2 cell line. The anticancer activity exerted by H1, H2, and H3 was not significantly different. This anticancer activity of H, H2, and H3 increased significantly (0.02, 0.03, and 0.02, respectively) with the presence of AgNPs. H1 showed anticancer.

Honey as a traditional medicine and dietary natural product has recently become the focus of attention in the treatment of certain diseases as well as promoting overall health and well‐being. There is strong evidence supporting the positive role of natural food and food product on the induction of apoptosis in different tumor cells (Samarghandian et al. 2011). In this regard, we investigated the antiproliferative honey kinds on Hela and HepG2 cell lines. We found that honey was cytotoxic toward the cancer cells. Some worker (Sadeghi‐Aliabadi et al., 2015) showed the same result when tested on HepG2 cell line as the IC50 was 3.12% and this effect was dose dependent. In the oral health setting, honey has been found to be effective for the treatment of radiation‐induced oral mucositis (Biswal, Zakaria, & Ahmad, 2003) and is also found to be anticarcinogenic (Sela, Maroz, & Gedalia, 2000) and antiproliferative and induces apoptosis in prostate cancer cells (Samarghandian et al., 2011). This is consistent with results being presented in this study. The honey tested in this study has been originally produced in Saudi Arabia (Rijal Ulma, Aseer, Saudi Arabia) and Pakistan, and sold as Sidr honey in the local markets. According to our best knowledge, Sidr honey has not been reported to be tested against Hela and HepG2 cancer cells. Some researchers (Attia, Gabry, El‐Shaikh, & Othman, 2008; Gribel' & Pashinskiĭ, 1990) reported that honey revealed moderate antitumor and pronounced antimetastatic effects. Their results also showed that the antitumor activity of 5‐fluorouracil and cyclophosphamide has been increased in combination with honey. Honey inhibited the growth of bladder cancer cell lines in vitro, and bladder cancer antiproliferative activity of honey may relate to its low pH (3.2–4.6). It was suggested that the polyphenols found in honey, including caffeic acid, and its phenyl esters, present in natural honey at the levels of 20%–25%, to be promising pharmacological agents in the treatment of cancer by reviewing their antiproliferative and molecular mechanisms (Jaganathan & Mandal, 2009). These compounds are thought to exhibit a broad spectrum of activity including tumor inhibition (Rao et al., 1993) by downregulation of many cellular enzymatic pathway including protein tyrosine kinas cyclooxygenase and ornithine decarboxylase pathways (Rao et al., 1993). Jungle honey obtained from the tropical forest of Nigeria showed chemotactic activity for neutrophils, which were found to possess potent antitumor activity (Fukuda et al., 2011). Moreover, the expression of various proapoptotic and antiapoptotic proteins was found to be altered during apoptosis (Ghashm et al., 2010). Unfractionated honey induces cell‐growth arrest, resulting in cell cycle blockage at the sub‐G1 phase (Jaganathan & Mandal, 2010).

Studies have shown several possible mechanisms mediated the antiproliferative effect of honey toward cancer cells such as involvement in inducing antioxidant effects (Antony, Han, Rieck, & Dawson, 2000), stimulation of TNF‐α, involvement in the inhibition of lipoprotein oxidation (Swellam et al., 2003), and induction of apoptosis and cell cycle inhibition (Dai et al., 2013).

Regarding the induction of mild growth of Hela cells by honey, some authors considered honey as mitogenic agent (Tonks et al., 2001) where it enhances cell proliferation (Aljady, Kamaruddin, Jamal, & Yassim, 2000). Enhanced proliferation induced by honey was suggested to be a nutritional effect caused by the carbohydrate of honey which provides substrates for glycolysis. This does not contradict with its other antitumor effect (El‐Sayed et al., 2010). This idea was proved by extracting the sugar from honey (Aziz, Rady, Amer, & Kiwan, 2009).

## 5 CONCLUSIONS

Sidr (*Ziziphus* *spina‐christi*) honey was collected from three different geographical locations, two from KSA and one from Pakistan. All collected honeys could produce AgNPs from silver nitrate solution in a spherical shape. Sidr honey collected from KSA and produced by *Apis mellifera jemenitica*, alone or containing AgNPs, stimulated the growth of normal rat splenic cells while both Sidr honey collected from Pakistan and KSA and produced by *Apis florea* inhibited the splenic cells' growth. All honeys alone stimulated Hela cancer cell line, but inhibited its growth when contained AgNPs. All honeys, alone or containing AgNPS, inhibited HepG2 cancer cell line proliferation. All honeys showed antimicrobial activities, these activities increased when honeys contained AgNPs. Sidr honey can be used for antimicrobial agent, but can be used as anticancer agent with care as it stimulated cell growth of some lines (e.g., Hala) and inhibited another (e.g., HepG2).

## CONFLICT OF INTEREST

All authors state that they have not any financial/commercial conflict of interest regarding this work.

## ETHICAL APPROVAL

This study was approved by the Ethical Committee of King Khalid University.
